# Nutrition for brain health: Keeping adolescents in MIND

**DOI:** 10.1038/s41390-024-03095-6

**Published:** 2024-03-19

**Authors:** Syeda-Samar Sohail, William B. Mitchell

**Affiliations:** Department of Pediatrics, Albert Einstein College of Medicine, Bronx, NY USA

***Commentary*** *to:* Social epidemiology of the Mediterranean-Dietary Approaches to Stop Hypertension Intervention for Neurodegenerative Delay (MIND) Diet among early adolescents: the Adolescent Brain Cognitive Development Study.

The Mediterranean-Dietary Approaches to Stop Hypertension Intervention for Neurodegenerative Delay (MIND) Diet was developed to promote brain health in the elderly.^1–3^ This diet combines the Mediterranean and the Dietary Approach to Stop Hypertension (DASH) diets, simplifying them into 10 brain-healthy food groups to consume and 5 unhealthy food groups to avoid.^4^ The Mediterranean diet has been shown to protect against the occurrence of several adverse health outcomes and reduce the incidence of cancer, cardiovascular disease, neurodegenerative disease, and mortality among the adult population. Several studies have looked at the effects of Mediterranean diet on adolescents and have demonstrated a positive association with decreased obesity, improved academic performance and improvement of chronic diseases.^5–7^ The DASH diet was designed to reduce hypertension and to lower the risk of cardiovascular disease.^8,9^ The DASH diet has been associated with improved blood pressure and may be protective against gastroesophageal reflux disease in adolescents.^10,11^ For example, significant benefits have been seen in blood pressure and lipid profiles in adolescents with hemophilia.^12^

Adolescence is a crucial period of physical growth and neurological development, and nutrition plays a very important role in that development. Given its impact on neurocognitive function in the elderly, there is reason to anticipate that the MIND diet could benefit the developing brains of adolescents as well. In this age group, a healthy and balanced nutritional diet is an important preventive element of risk factors of disease development, especially for chronic diseases such as obesity, diabetes, hypertension, and dyslipidemia.^13^ Prior studies have found poor adherence of adolescents to the recommended dietary guidelines, including the Mediterranean diet, in the United States.^14^ Uptake of nutritional recommendations is clearly affected by sociodemographic disparities affecting adolescents. Previously documented disparities, including race, household income and food security status are known risk factors for poor nutrition.^15,16^ Against this backdrop, in this issue of Pediatric Research, Nagata and colleagues examine the relationship between sociodemographic factors and adherence to the MIND diet in early adolescents.

The MIND diet has been a success in the older adult population. Adherence to this diet in older adults is associated with better cognitive function, slower cognitive decline, decreased risk of dementia, and fewer depressive symptoms.^1–3^ One pediatric study has shown a reduced risk of obesity in school-aged children who adhered to the MIND diet,^17^ but the uptake and efficacy of this diet in pediatrics is largely unknown. Building on this nutritional literature, the study by Nagata and colleagues represents a significant effort to examine the uptake of the MIND diet in early adolescents.

To evaluate the relationship of MIND diet uptake and sociodemographic factors, the authors created a dietary history database to examine the diet patterns of over 8000 nine to twelve-year-olds. This cohort of subjects was extracted from the database of the Adolescent Brain Cognitive Development (ABCD) study cohort. The ABCD study is a ten-year longitudinal effort to capture biobehavioral, environmental, familial, and genetic open-access data from over 11,000 adolescents throughout the United States. For the current study, the investigators had each child and their caregiver complete a derivative of the MIND diet questionnaire, which measures both adherence to the MIND diet and overall nutritional status. They also looked at sociodemographic characteristics including sex at birth (male or female), race/ethnicity (Asian, Black, Latino, Native American, White, or other), annual household income, highest parent education level, age and sexual orientation. Multivariable linear regression analysis was conducted to investigate the associations between sociodemographic factors and MIND adherence measured via the total nutrition score.

While the study found uptake of the MIND diet by adolescents to be similar to that of older adults, there were significant sociodemographic differences in uptake. Some differences were in line with the findings of prior nutritional diet uptake studies in children,^15,16^ such as higher uptake in female sex, Latino ethnicity, Asian and Black race, and higher household income. A notable addition to the literature is the finding that heterosexual sexual orientation was associated with a higher adherence to the MIND diet in adolescents. These findings reinforce prior nutrition-related findings in this subgroup, which include high rates of obesity, eating disorders and food insecurity in adolescent sexual minorities.^18–20^ This is one of the first large clinical trials to include adolescent sexual minorities as a sociodemographic subset, and specifically to document diet in adolescents who are sexual minorities.^18^ This important aspect of the study highlights a gap in the literature, namely the inclusion of sexual minority groups as sociodemographic cohorts, as well as highlighting the need for the implementation of nutritional health interventions in adolescent sexual minorities.

In summary, given the positive effect of the MIND diet on brain health in the elderly, it is exciting to look forward to potential studies of the uptake of the MIND diet and brain health outcomes of this cohort of adolescents. The source of the data, namely the longitudinal ABCD study, will collect multifactorial brain health data over ten years. Therefore, this research team is now poised to generate a ten-year longitudinal study of the uptake of the MIND diet in adolescents, further delineating the uptake of the MIND diet on vulnerable groups of adolescents. Drawing on data from the ongoing ABCD study, future studies could also describe the longitudinal impact of the MIND diet on brain health in adolescents. These findings could, in turn, give rise to opportunities for clinically meaningful nutritional interventions for at-risk adolescents.
